# Collectanea Medica: Consisting of Anecdotes, Facts, Extracts, Illustrations, &c.

**Published:** 1822-12

**Authors:** 


### COLLECTANEA MEDICA:

CONSISTING OF

ANECDOTES, FACTS, EXTRACTS, ILLUSTRATIONS, &c.

*Relating to the History or the Art of Medicine, and the Collateral Sciences.*

Floriferis, ut apes, in saltibus omnia libant,
Omnia nos, itidem, depascimur aurea dicta.

---

ART. I.—*An Account of the last Illness, Decease, and Post Mortem Appearances of Napoleon Bonaparte.* By ARCHIBALD ARNOTT, M.D. Surgeon 20th Regiment.

BEFORE I visited Napoleon Bonaparte, I was consulted upon his case on the 25th of March, by his own medical attendant, Prof. Antomarchi, who stated to me that Napoleon Bonaparte had long been labouring under some great derangement of function in the digestive organs, which was characterized by gastrodynia, nausea, and vomiting, especially after taking food, very obstinate costiveness, and great wasting of flesh and strength. He further mentioned, that, on the 17th of that month (March), Napoleon Bonaparte had been seized with a febrile attack, which he (Professor Antomarchi) in Italian termed *febbre gastrica pituitosa.* He informed me that he had administered an emetic, cathartics, and antimonials in small doses, with the view of determining to the surface at the onset of the fever: however, he said, the symptoms were still urgent,—viz. increased heat, great prostration of strength, pain in the epigastric region, most distressing vomiting, and constipated bowels.

He passed a good night betwixt the 19th and 20th of April, only that, from eleven to three o'clock, he was somewhat teased with a sensation of heat in his bowels, and of choking, accompanied with thirst; and, when he drank any thing, it was with difficulty he could swallow: this is as he expressed it. In the morning he was very composed; heat natural; the stomach had retained every thing he had eaten since the day before; and he had had a good alvine evacuation from an enema at nine that morning. Towards evening, however, he complained of a pain and heat about the scrobiculus cordis; said he had a continual nausea, and that it was only by keeping himself very quiet he did not vomit: yet the little nourishment he took rested on his stomach.

On the 21st of April, Professor Antomarchi's report to me was, "The Emperor has passed a good night; he has eaten twice in the night without vomiting any: this morning, however, at seven o'clock, he vomited a little of what he had eaten." I found him very composed, and he told me he was free from pain. His pulse near 75; state of the skin and heat quite natural. He had two very good alvine evacuations that morning from an enema.

On the morning of the 22d of April, he felt a sensation of heat, with dryness in the stomach, and a sense of suffocation; had vomited a little the night before, and likewise some before I saw him that day. What came off his stomach was shown to me,—it was a something he had eaten the day before, quite undigested. After the vomiting, his stomach was much relieved, and he conceived himself better on the whole: he had passed a very good night, and had slept several hours. He on that day referred all his disease to the stomach. He had a small dejection from an enema. The urine deposited a lateritious sediment. Pulse was 84, and more feeble. For the purpose of gently exciting the action of the primæ viæ, we prevailed on him that day to take a weak solution of sulphate of magnesia, in an infusion of gentian, with the addition of a little tincture of the same, according to the following form: —℞. Magnesiæ Sulphatis ʒ vj. solve in Aquæ Puræ octa. adde Infus. Gentianæ compositæ ℥ vj. et Tinct. Compositæ ejusdem ℥ss. m. f. mistura, cujus sumat cochlearia tria ampla subinde.

23*d April.*—Pulse was 78, and heat natural; had a small dejection from an enema that morning; had vomited twice since the former visit. He had taken the medicine ordered, and it had rested on his stomach; he thought himself stronger, and we ordered him to continue the medicine and diet as before. He continued tolerably easy all the day of the 23d, and took a little light nourishment about seven in the evening; he, however, vomited it soon afterwards. He had some accession of fever at nine, but upon the whole was easy; slept the greatest part of the night; and on the morning of the 24th there was a clear remission of all the febrile symptoms; the pulse was at 78, and heat natural. He had a copious alvine evacuation that morning, but complained of great weakness and of giddiness: we gave directions to go on as before. He was very ill on the night between the 24th and 25th; the vomiting was incessant, and he had no sleep whatever. In the morning, his strength was much sunk; the pulse was 82, and weak. He could not be advised to continue the medicine, but had a small dejection that morning from an enema. The vomiting continued, with little intermission, throughout the whole day of the 25th; the stomach rejected every thing that was swallowed. Observing that, when the primæ viæ were open, the nausea and vomiting were less violent, I urged him to take some aperient medicine; and, being convinced he had derived benefit from the solution of the sulphate of magnesia in the infusion of gentian, I pressed him to take a full dose of that medicine, and accordingly he took it that morning. It emptied the bowels well: it had not, however, the usual good effect of lessening the vomiting; that distressing symptom continued. He had an evening accession of fever, was restless, and raving at times until three o'clock in the morning of the 26th. He then went to sleep, and did not awake until seven: soon after that he was seized again with violent vomiting. What came off the stomach was something undigested, mixed with phlegm of a ropy consistence.

On the morning of the 26th, the pulse was 86; heat lower than natural, with a cold, damp, clammy perspiration all over him. He complained much of pain in the hypochondriac and epigastric regions, which he referred to his stomach and liver. He on that day asked me what was to be done for him, and what I considered his disease to be? The symptoms, I told him, I thought indicated some great derangement in the digestive organs. He put the question to me again that day, if I supposed his liver affected? My reply was, that I did not think there was any diseased structure of that viscus; perhaps there might be a want of due action in it. Professor Antomarchi and myself both agreed that the same mode of treatment should be pursued.

On the morning of the 27th of April, our patient was low and comatose, had passed a restless night, and had had frequent fits of vomiting. I had not been long at his bed-side before he was again seized with violent retching and vomiting; and, on examining the basin, I observed that what had come off the stomach was a dark-coloured fluid resembling coffee-grounds, and very offensive. That vomiting continued until half after three o'clock P.M.; it then ceased, and he went to sleep. Pulse kept at 84, and heat about natural. In the evening he was more tranquil, free from pain, but low and exhausted. He would not take any medicine, and, as the first passages had not been emptied for forty hours, we ordered an enema; we also wished to apply a blister to the region of the stomach, but this he likewise objected to; on which we proposed a warm stimulating plaster, which he consented to, and we accordingly applied one of the emplast. aromaticum, of the Dublin Pharmacopœia.

On the morning of the 28th, I was informed by his attendants that he had been vomiting frequently in the course of the night; that he still continued to do so; and what came off the stomach had the same appearance as it had the day before: it was a dark-coloured grumous fluid, and contained small specks of blood. Pulse was 84 and weak, and there appeared great exhaustion. He talked incoherently; had no alvine evacuation from the last enema; yet he most pertinaciously refused to take medicine, and would not allow even another enema to be administered. Towards evening, however, the bowels becoming very uneasy, he was prevailed upon to allow one to be administered, which procured two small evacuations; yet he passed a sleepless night, raved greatly, but had less vomiting, and what came off the stomach was not so dark-coloured.

He had three hours' sleep on the morning of the 29th: when he awoke he was sensible and composed, the pulse 87. Sometime during the night, in a raving fit, he had torn the emp. aromaticum off, but consented to have a blister applied over the stomach, which was done forthwith; and, with the view of tending to lessen that dreadful irritation of stomach, we prescribed the following:

℞. Aquæ Menthæ Virid. ℥iss.

Potassæ Subcarbonatis, ℈j.

Succi Lim. Recentis q. s. ad saturand.

Tincturæ Calumbæ, minima xxx.

——— Opii, minima v. Misce ut fiant haustus 6ta quaque hora sumendus.

*30th April.*—The blister over the stomach had risen, and in the night Professor Antomarchi had applied one to the inside of each thigh. The draughts were not taken as ordered. He slept for some hours in the beginning of the night, and towards morning, although he did not sleep, he lay quiet and composed. He vomited several times, but not so much, nor was the matter that came off the stomach of such a dark colour as before; heat was natural and general; pulse 90 and regular; intellect was more collected; his respiration easy, and he lay in a composed state. Count Montholon informed me that he had singultus for two hours during the night. Had had no alvine evacuation, nor could he be advised to use any means to procure one. Betwixt eleven and twelve o'clock that night, our patient was seized with a rigor, accompanied with great anxiety and dyspnœa, and followed by singultus.

On the morning of the 1st of May, he was much worse, his strength had sunk considerably, there appeared more anxiety than usual about him; the pulse had become more frequent, the skin clammy, the heat below natural; he had strong singultus, and talked incoherently. An enema was administered, which produced a copious evacuation.

On the morning of the 2d of May, there was an aggravation of all the symptoms; almost continued singultus, anxiety, restlessness, and quick and oppressed respiration. The heat was natural and equable, the extremities keeping warm. Had some retching and vomiting. Pulse 102 and small, and in the evening rose to 108. He went to sleep at ten o'clock that night, and did not awake until three next morning: he was then insensible, and showed great anxiety and restlessness. Pulse 100, small and weak; had no vomiting since the night before, and then it was inconsiderable. Singultus became very strong and distressing, the delirium increased, and he began to articulate very indistinctly. Having had no alvine evacuation since the morning of the 1st, an enema was proposed, but our patient was so unmanageable that it could not be administered. In the course of that day, (3d May,) all the symptoms became more aggravated; added to which, a fulness and tension of the belly came on. I then conceived it indispensable to free the primæ viæ by some means, and, being aware of our patient's aversion to medicine, I recommended a dose of calomel to be given, which might be done without his knowledge: accordingly, ten grains of that medicine were given at six o'clock that evening. The calomel commenced its operation at half after eleven, five hours and a half after it was taken, and before noon next day it had produced five copious alvine evacuations, exactly resembling tar in colour and consistence, and remarkably fetid. After this he was somewhat relieved; there was less restlessness and anxiety; he was more sensible to objects around him, but was in a state of great debility; the pulse was small, weak, and easily compressed. The singultus continued, and, with the view of relieving it, we gave a draught of tinctur. opii et spiritus æther. vit.; and, to support his strength, he took a little jelly and wine occasionally.

At this time milk was particularly recommended to him, but it was objected to. I left him at nine o'clock that evening (4th May,) in a sound sleep, breathing easy; and I was informed by those who were watching him, that he was tolerably composed and easy during the night, and until five o'clock in the morning; he was then seized with vomiting, and a dejection passed involuntarily. I was called immediately, and, on examining the matter that had come off the stomach, I found it resembled the dark-coloured fluid which he had vomited on the 27th April. He had then great dyspnœa; there was a total loss of muscular motion, the under jaw had dropped, and he had lost the power of deglutition; the eyes were fixed, the pulse varied from 102 to 110 in the minute, was small and weak, intermitted, and was easily compressed. That nothing should be left undone, although *moribundus*, sinapisms were applied to the feet, blisters to the legs, and one to the sternum, but none of them took effect; and all the symptoms increased until eleven minutes before six o'clock P.M. when he expired.

*Appearances on Dissection of the Body of Napoleon Bonaparte.*

On a superficial view, the body appeared very fat, which state was confirmed by the first incision down its centre, where the fat was upwards of one inch thick over the sternum, and one inch and a half over the abdomen.

On cutting through the cartilages of the ribs, and exposing the cavity of the thorax, a trifling adhesion of the left pleura to the pleura costalis was found; about three ounces of reddish fluid were contained in the left cavity, and nearly eight ounces in the right.

The lungs were quite sound.

The pericardium was natural, and contained about an ounce of fluid.

The heart was of the natural size, but thickly covered with fat; the auricles and ventricles exhibited nothing extraordinary, except that the muscular parts appeared rather paler than natural.

Upon opening the abdomen, the omentum was found remarkably fat; and, on exposing the stomach, that viscus was found the seat of extensive disease: strong adhesions connected the whole superior surface, particularly about the pyloric extremity, to the concave surface of the left lobe of the liver; and, on separating these, an ulcer, which penetrated the coats of the stomach, was discovered one inch from the pylorus, sufficient to allow the passage of the little finger. The internal surface of the stomach, to nearly its whole extent, was a mass of cancerous disease, or schirrous portions advancing to cancer: this was particularly noticed near the pylorus. The cardiac extremity, for a small space near the termination of the œsophagus, was the only part appearing in a healthy state. The stomach was found nearly filled with a large quantity of fluid, resembling coffee-grounds.

The convex surface of the left lobe of the liver adhered to the diaphragm; but, with the exception of the adhesions occasioned by the disease in the stomach, no unhealthy appearance presented itself in the liver.

The remainder of the abdominal viscera were in a healthy state. A slight peculiarity in the formation of the left kidney was observed.*

---

* We have been induced to give the above extracts from the pamphlet lately published by Dr. ARNOTT, because, independent of the interest attached to every thing connected with Bonaparte, it forms a good case of cancer of the stomach.

